# Metformin Alleviates Hepatic Steatosis and Insulin Resistance in a Mouse Model of High-Fat Diet-Induced Nonalcoholic Fatty Liver Disease by Promoting Transcription Factor EB-Dependent Autophagy

**DOI:** 10.3389/fphar.2021.689111

**Published:** 2021-07-23

**Authors:** Dan Zhang, Yicheng Ma, Jianjun Liu, Yi Deng, Bo Zhou, Yu Wen, Mingke Li, Daiyan Wen, Yunyan Ying, Sufeng Luo, Chunjing Shi, Guangyu Pu, Yinglei Miao, Chenggang Zou, Yuanli Chen, Lanqing Ma

**Affiliations:** ^1^ The First Affiliated Hospital, Yunnan Institute of Digestive Disease, Yunnan Clinical Research Center for Digestive Diseases, Kunming Medical University, Kunming, China; ^2^ State Key Laboratory for Conservation and Utilization of Bio-Resources in Yunnan, School of Life Sciences, Yunnan University, Kunming, China; ^3^ Yunnan Key Laboratory of Stem Cell and Regenerative Medicine, Research Center of Biomedical Engineering, Kunming Medical University, Kunming, China; ^4^ Faculty of Basic Medicine, Kunming Medical University, Kunming, China

**Keywords:** metformin, nonalcoholic fatty liver disease, autophagy, TFEB, hepatic steatosis, insulin resistance

## Abstract

Nonalcoholic fatty liver disease (NAFLD) results from an abnormal accumulation of lipids within hepatocytes, and is commonly associated with obesity, insulin resistance, and hyperlipidemia. Metformin is commonly used to treat type 2 diabetes mellitus and, in recent years, it was found to play a potential role in the amelioration of NAFLD. However, the mechanisms underlying the protective effect of metformin against NAFLD remain largely unknown. Transcription factor EB (TFEB) is a master transcriptional regulator of lysosomal biogenesis and autophagy and, when activated, is effective against disorders of lipid metabolism. However, the role of TFEB in hepatic steatosis is not well understood. In this report, we demonstrate that the activity of TFEB is reduced in the liver of mice fed a high-fat diet. Metformin treatment significantly reverses the activity of TFEB, and the protective effect of metformin against hepatic steatosis and insulin resistance is dependent on TFEB. We show that metformin-induced autophagy is regulated by TFEB, and our findings reveal that TFEB acts as a mediator, linking metformin with autophagy to reverse NAFLD, and highlight that TFEB may be a promising molecular target for the treatment of NAFLD.

## Introduction

Nonalcoholic fatty liver disease (NAFLD) is a major liver disease subtype and an increasingly recognized cause of other liver-related diseases. NAFLD, which is characterized by an excessive hepatic lipid accumulation, covers a wide range of liver disorders, including steatosis, steatohepatitis, fibrosis, and cirrhosis (Sliz et al., 2018). With the growing epidemics of diabetes, obesity, and other metabolic syndromes, the prevalence of NAFLD is dramatically increasing worldwide. The classical pathogenesis of NAFLD is based on the “two-hit hypothesis” (Day and James, 1998; Dowman et al., 2010); however, the molecular mechanisms underlying the pathogenesis of NAFLD need to be explored. A growing number of studies have implicated that the pathogenesis of NAFLD, in which some risk factors, including lipid metabolic disorders, chronic inflammation, and oxidative stress, play a central role, is complex (Krawczyk et al., 2010). Due to the complex multi-hit pathogenesis of NAFLD, there are no effective pharmacological therapies for it. Thus, new therapeutic strategies that reverse or prevent lipogenesis, thereby exerting indirect effects on NAFLD need to be developed.

Metformin, a widely used agent for noninsulin-dependent diabetes, has therapeutic potential to restore glucose and lipid metabolic homeostasis (Knowler et al., 2002; Foretz et al., 2014). Although metformin has been used as an effective drug for the treatment of type 2 diabetes for several decades (Han et al., 2017), its hypoglycemic mechanism remains controversial. Accumulating evidence has demonstrated that metformin inhibits hepatic gluconeogenesis and improves the peripheral utilization of glucose by blocking the mitochondrial respiratory chain complex I (Viollet et al., 2012; Flory and Lipska, 2019). More recent studies found that metformin can activate AMP-activated protein kinase (AMPK) (Maida et al., 2011), which in turn reduces hepatic lipogenesis and exerts an indirect effect on hepatic insulin sensitivity to control the hepatic glucose output (Viollet et al., 2012).

Recent studies have reported a novel role of metformin in improving the healthspan and extending the lifespan in several animal models, such as mice and Caenorhabditis elegans (Anisimov et al., 2008; Martin-Montalvo et al., 2013; De Haes et al., 2014; Chen et al., 2017). The benefits of metformin in attenuating the aging process are mainly associated with augmented autophagy, which is primarily mediated by AMPK, mammalian target of rapamycin (mTOR), NAD-dependent deacetylase sirtuin-1 (SIRT1), and insulin/IGF-1 signaling (IIS) pathways (Kim et al., 2011; Shi et al., 2012; Chen et al., 2016; Cuyas et al., 2018; Kulkarni et al., 2020). Therefore, the specific mechanisms underlying metformin-induced autophagy have received growing attention in recent years.

Transcription factor EB (TFEB) belongs to the bHLH-leucine zipper transcription factor family, which serves as a major transcription factor of lysosome- and autophagy-related genes (Sardiello et al., 2009; Settembre et al., 2011). The activity of TFEB is closely related with its phosphorylation status. The phosphorylated TFEB is the inactive form and is mainly located in the cytoplasm. Under stress conditions, TFEB is activated *via* kinase inactivation- and phosphatase activation-mediated dephosphorylation and is translocated into the nucleus (Settembre et al., 2012; Li et al., 2019). TFEB is a critical factor in the response to glucolipotoxicity, which results from obesity and diabetes (Trivedi et al., 2016). TFEB can promote the expression of peroxisome proliferator-activated receptor γ coactivator 1α (PGC1α), resulting in increased lipid degradation, fatty acid oxidation, and decreased lipogenesis (Settembre et al., 2013; Evans et al., 2019). Thus, TFEB plays a key role in lipid metabolism. However, whether TFEB is involved in the protective effect of metformin against NAFLD has not yet been reported.

In this study, we demonstrated that metformin treatment improved hepatic lipid accumulation and insulin resistance (IR) in HFD-fed mice. The protective effect of metformin is probably associated with the activation of autophagy, which was also activated by metformin. Finally, we showed that metformin-induced autophagy was mediated by TFEB. Taken together, these observations suggest that metformin exerts a potential protective effect against NAFLD, whose molecular mechanism is associated with TFEB-dependent increased autophagy.

## Materials and Methods

### Animals

Adult (6 wk old) C57BL/6J (male, 21–23 g) were obtained from the Nanjing Biomedical Research Institute of Nanjing University (License no. SCXK [S] 2005-0019). The animal procedures and treatments conformed to the Guide for the Care and Use of Laboratory Animals of China National Institutes of Health. The mice were housed in standard experimental cages and allowed free access to food and water at 20°C ± 2°C and at a humidity of 50% ± 5%.

### Reagents and Antibodies

Metformin (Met, C4H11N5·HCl; purity ≥97%), was provided by Sigma-Aldrich (D150959; Shanghai, China), and dissolved in sterile saline. Isoflurane for inhalation was purchased from RWD Life Science Co, Ltd (R510-22-4; Shenzhen, China). A high-fat diet consisted of 20% carbohydrate, 20% protein, and 60% fat (total 25.07 kJ/g), which was purchased from Beijing Botai Hongda Biotechnology (HD004; Beijing, China). Mouse INS (Insulin) ELISA Kit and TG assay Kit were acquired from Elabscience (E-EL-M1382c, E-BC-K261; Wuhan, China). H&E Staining Kit and Oil-red O Staining Kit were provided by Solar bio Science &Technology (G1120, G1261; Beijing, China). NE-PERTM Nuclear and Cytoplasmic Extraction Reagents were acquired from Thermo Fisher Scientific (78833; Waltham, MA, United States). Primary antibody against TFEB, Atg7, p62/SQSTM1 were purchased from Abcam (ab220695, ab133528, EPR4844; Discovery Drive, Cambridge Biomedical Campus, Cambridge, United Kingdom), LC3B was purchased from Cell Signaling (#83506; Boston, MA, United States). The primary antibody against GAPDH and Histone-H3 were acquired from Servicebio (GB12002, GB11102; Wuhan, China). The ECL Plus Reagent Kit was purchased from Millipore (P90720; Bedford, MA, United States). RT First Strand cDNA Synthesis Kit and 2×SYBR Green qPCR Master Mix (High ROX) were provided by Servicebio (G3330, G3322; Wuhan, China).

### Study Design

**FIGURE 1:**
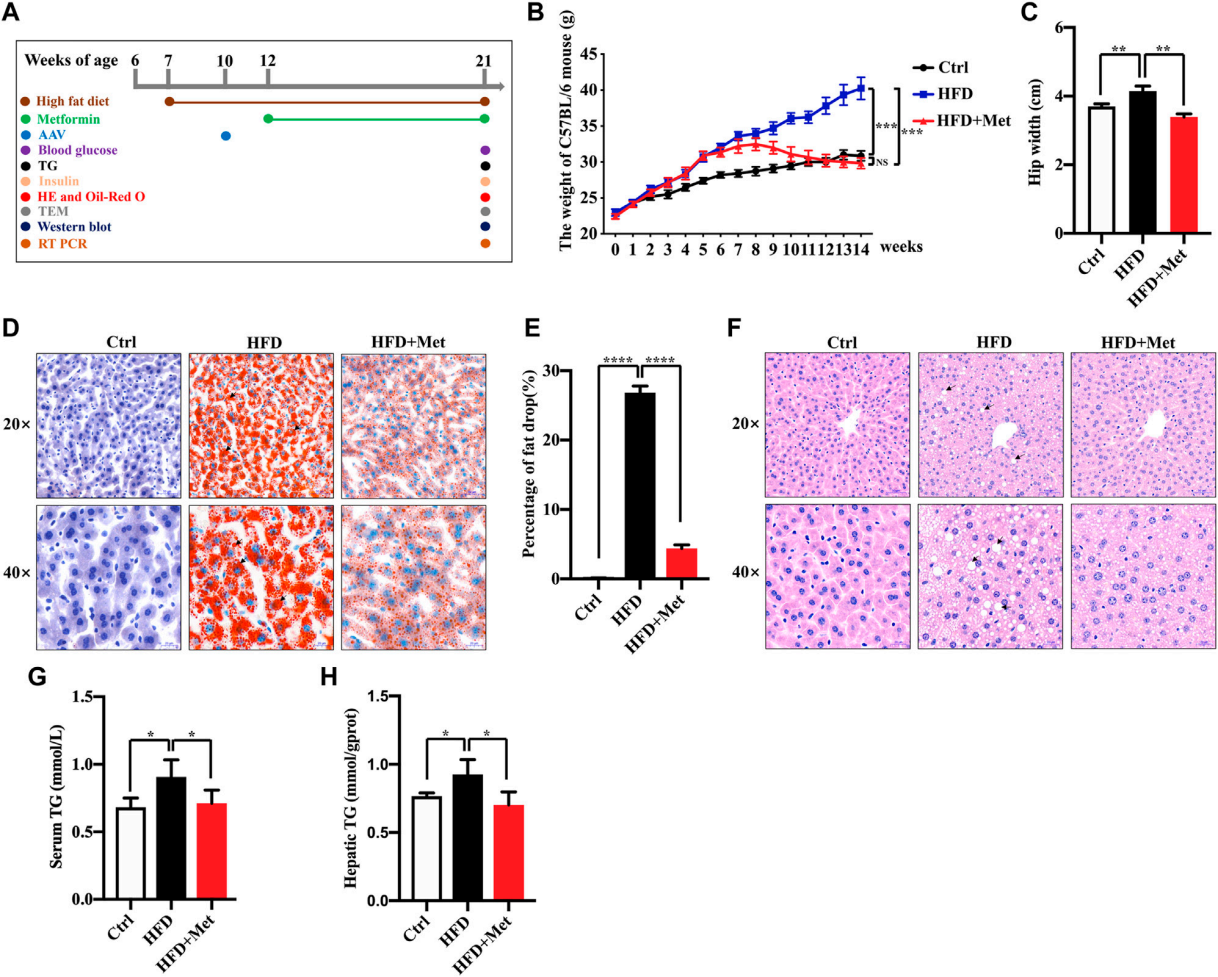
Effects of metformin on obesity and hepatic steatosis in HFD-fed mice. **(A)** Experimental scheme. **(B**,**C)** Effects of metformin on the body mass and hip width of mice. **(D)** Oil red O staining of the liver sections; representative images are shown, the arrows denote the fat drops (magnification ×200 or ×400; scale bar, 50 or 20 μm). **(E)** Quantification of the Oil red O staining intensity in the liver. **(F)** Representative pictures of H&E staining in the liver tissue, the arrows denote the fat vacuoles (magnification ×200 or ×400; scale bar, 50 or 20 μm). **(G)** Serum and **(H)** hepatic triglyceride (TG) levels in each group. Values are presented as mean ± SEM, *n* = 6 per group. *p < 0.05, **p < 0.01, ***p < 0.001, ****p < 0.0001, HFD group versus the control group, or metformin + HFD-fed group versus the HFD group.

The mice were divided randomly into five groups (*n* = 6 per group): the control group (the mice were fed a normal diet); high-fat diet (HFD)-fed group (the mice were fed a high-fat diet for 14 wk, and were orally gavaged with sterile saline daily for the last 9 wk); HFD + Metformin (Met) group (the HFD-fed mice were orally gavaged with metformin (300 mg/kg) daily for the last 9 wk) (Figure 1A); and the HFD + Met + Scramble control group and the HFD + Met + TFEB shRNA group (the mice received 100 μl of viral vectors (1.00 × 10^11^ viral genomes) *via* tail vein injections after 3 wk of HFD feeding) (Figure 1A).

### RNA Interference

For TFEB inhibition, knockdown experiments were performed *via* tail vein injection of adeno-associated virus serotype-8 (AAV8) carrying shRNA against TFEB (shRNA sequence of the TFEB gene: 5′-GCG​GCA​GTA​CTA​TGA​CTA​TGA​T-3′) or a scrambled shRNA control. Recombinant AAV8-shRNA-TFEB and its scrambled control were both constructed and packaged by Ubigene Biosciences (Guangzhou, China).

### Histology

Liver tissues were embedded in an OCT solution, frozen in liquid nitrogen, and cut into 5 μm sections. For the Oil red O experiment, the sections were stained with an Oil red O staining solution for 10 min at 60°C, and then rinsed with 60% isopropyl alcohol, re-stained with hematoxylin, and mounted. For hematoxylin and eosin (H&E) staining, the liver sections were stained with H&E according to the standard H&E protocol. Images were acquired using a Leica aperio CS2 system and then analyzed using ImageJ software.

### ELISA

Blood was collected from the heart, and the fasting blood glucose level was measured. The blood samples were left at room temperature for 2 h, and then centrifuged at 1,000 × *g* for 20 min. The supernatant was collected, and the serum insulin content was detected using an INS ELISA kit (E-EL-M1382c; Elabscience, Wuhan, China). Finally, the homeostatic model assessment for insulin resistance (HOMA-IR) index and the homeostatic model assessment for insulin sensitivity index (HOMA-ISI) of each group were calculated using the following formulae (Fang et al., 2010): insulin resistance index = [fasting blood glucose (mmol/L) × serum insulin (mIU/L)] / 22.5; insulin sensitivity index = 1 / [fasting blood glucose (mmol/L) × serum insulin (mIU/L)]. Triglycerides (TGs) in the liver tissue or serum were measured using a TG assay kit (E-BC-K261; Elabscience, Wuhan, China).

### Western Blotting

Fresh liver tissues were homogenized in liquid nitrogen, and the cytosolic and nuclear extracts were isolated using NE-ER™ Nuclear and Cytoplasmic Extraction Reagents (78833; Thermo Fisher, United States), following the manufacturer’s instructions for the quantitative determination of TFEB protein. Total cell extracts were collected, and high-efficiency RIPA lysate was used to detect the expression of autophagy-related proteins. The protein extracts were loaded into each well (20 μg/10 μl) and separated using sodium dodecyl sulfate-polyacrylamide gel electrophoresis (SDS-PAGE) on a 10 or 12.5% acrylamide gel, and then the proteins were transferred onto polyvinylidene fluoride (PVDF) membranes (P90720; Millipore, Bedford, MA, United States) and blocked with 5% skim milk-TBST for 2 h at 20°C. Then, the blots were incubated with primary antibodies (anti-TFEB 1:500, anti-ATG7 1:25,000, anti-p62/SQSTM1 1:20,000, anti-LC3B 1:1,000, anti-GAPDH 1:2,000, and anti-histone-H3 1:5,000). After being washed and incubated with the secondary antibodies, the blots were imaged using an imaging system (Amersham Imager 600) and densitometric analysis of the Western blot signals as of the 16-bit.jpg digital images of the blots were performed using ImageJ (NIH). Briefly, the images were converted to grayscale, rectangles were drawn around the bands, a profile plot was generated for each band, and the density of each band was determined based on the area under the peak by using the wand tool. Finally, the relative density value of target protein was calculated by dividing its density value by the control (GAPDH or H3) density value. Moreover, the final value of target protein was obtained by dividing the relative value in the experimental group by that in the control group.

### Transmission Electron Microscopy

Fresh liver tissues (1 mm^3^ of volume) were fixed using pre-chilled electron microscopy fixative and then with 1% osmium tetroxide for 2 h, and stained with 0.5% uranyl acetate in a 50 mM maleate buffer (pH 5.15) for 30 min. After dehydration in ethanol, the tissues were treated for 1 h in propylenoxide, and then embedded in Epon/Araldite resin. Ultrathin sections were cut and mounted on copper grids. Autophagosomes were imaged using an HT7700 transmission electron microscope (Hitachi High Technologies, Tokyo, Japan). The autophagosomes per section were counted and normalized by the surface area.

### Quantitative Real-time PCR (qRT-PCR)

Total RNA was isolated using Trizol reagent (10296010; Invitrogen, Camarillo, CA). cDNA was generated using an RT First Strand cDNA Synthesis kit (G3330; Servicebio, Wuhan, China). qRT-PCR was performed using the SYBR Premix Ex Taq (Takara) and a Roche LightCycler 480 System (Roche Applied Science, Mannheim, Germany). The primers used for the qRT-PCR assay are listed in Supplementary Table 1. All qRT-PCR analyses were performed in triplicate. GAPDH was used for normalization, and the gene expression was analyzed according to the ΔΔCt method.

### Statistical Analyses

Statistical analyses were performed using GraphPad Prism 7 (GraphPad Software Inc., La Jolla, CA, United States). Data were expressed as mean ± standard error of the mean (SEM). The statistical significance of the differences was assessed using one-way ANOVA, followed by a Student-Newman-Keuls test. A value of *p* < 0.05 was considered statistically significant.

## Results

### Metformin Improves the Obesity, Hepatic Steatosis, and IR in NAFLD Mice

To investigate the effect of metformin against obesity, the HFD-induced obese (DIO) mice were treated daily with the vehicle (sterile saline) or metformin *via* oral gavage for 9 wk (Figure 1A). Metformin treatment drastically reduced the bodyweight and the average hip width of the DIO mice (Figures 1B,C). Hepatic steatosis is a hallmark of NAFLD. Thus, we determined the effect of metformin on the fat deposition in the liver by using both H&E and Oil red O staining. Our results showed that the hepatic lipid accumulation in DIO mice was significantly reduced by metformin (Figures 1D–F). Moreover, we found that the levels of serum and hepatic triglyceride (TG) were decreased in both the serum and liver of DIO mice after metformin treatment (Figures 1G,H).

**FIGURE 2:**
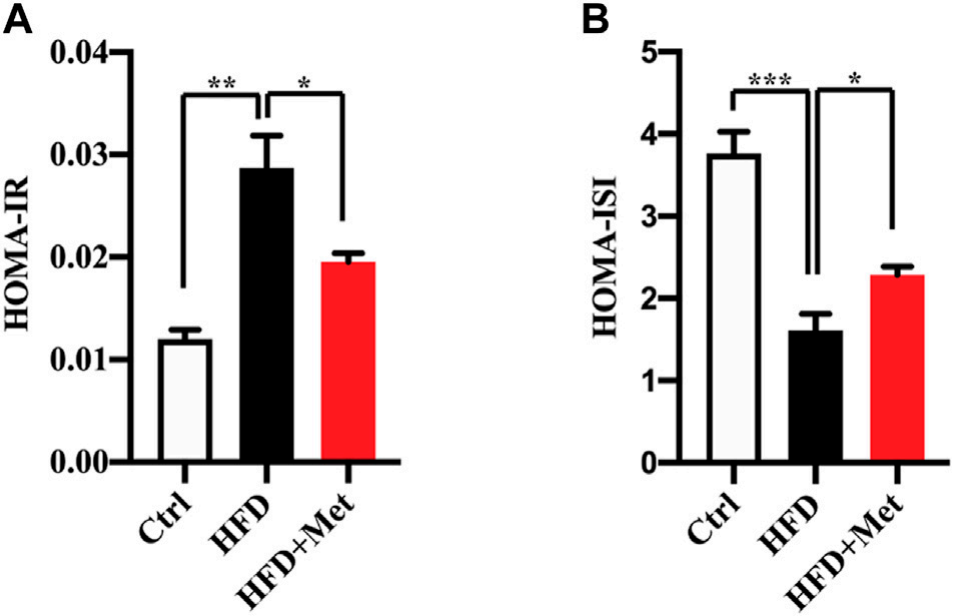
Effects of metformin on insulin resistance in HFD-fed mice. **(A)** HOMA-IR index and **(B)** HOMA-ISI in each group. Values are presented as mean ± SEM, *n* = 6 per group. *p < 0.05, **p < 0.01, ***p < 0.001, HFD group versus the control group, or metformin + HFD-fed group versus the HFD group.

NAFLD is closely associated with IR. We thus determined the effect of metformin on insulin resistance and found that the metformin treatment reduced the HOMA-IR (Figure 2A; Table 1) and increased the HOMA-ISI (Figure 2B; Table 1). Taken together, these results suggest that a metformin treatment significantly improves an excessive lipid accumulation, hepatic steatosis, and IR in the mice fed an HFD.

**TABLE 1:** The levels of fasting blood-glucose and insulin to calculate the HOMA-IR and HOMA-ISI after metformin treatment.

Group	Fasting blood-glucose (mmol/L)	Insulin (mIU/L)	HOMA-IR	HOMA-ISI
Ctrl	2.875 ± 0.350	0.096 ± 0.006	0.012 ± 0.001	3.763 ± 0.263
HFD	4.500 ± 0.844	0.151 ± 0.016	0.029 ± 0.003^a^	1.611 ± 0.198^b^
HFD+Met	4.900 ± 0.424	0.091 ± 0.005	0.020 ± 0.001^c^	2.285 ± 0.099^c^
*F*	3.403	0.955	2.958	0.626
*P*	0.079	0.421	0.103	0.557

Homeostatic model assessment for insulin resistance (HOMA-IR) index, Homeostatic model assessment for insulin sensitivity index (HOMA-ISI). Data presents mean ± SEM; n = 6.

a p < 0.01.

b p < 0.001, HFD group versus control group.

c p < 0.05, Metformin+HFD-fed group versus HFD group, respectively, using one-way ANOVA.

### Metformin Induces Autophagy in the Liver of NAFLD Mice

**FIGURE 3:**
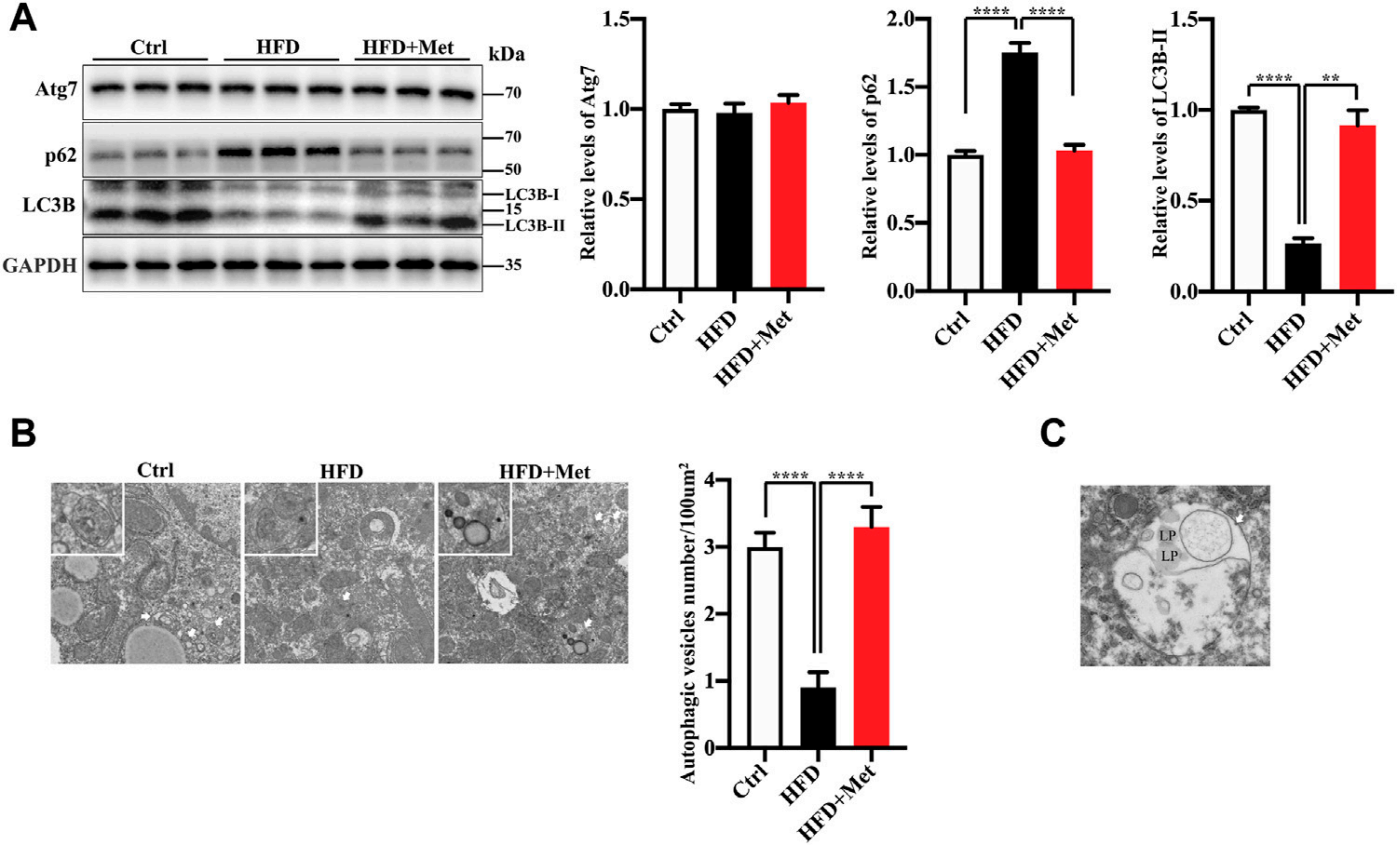
Metformin induces autophagy in the liver of HFD-fed induced NAFLD mice. **(A)** The protein levels of Atg7, LC3B-II, and p62 were determined using Western blot analysis. Representative Western blots are shown. **(B)** Transmission Electron Microscopy (TEM) images of autophagic vacuoles in hepatocytes. Representative electron microscopy images (magnification ×7,000; scale bar, 2.0 μm), the white boxes in the pictures represent the autophagic vesicles, including autophagosome or early autophagic vacuole and degradative autophagic vacuole. The autophagic vesicles (arrow) were quantified in each group. Values are presented as mean ± SEM, *n* = 6 per group. **(C)** TEM images of autophagic vacuoles (arrow) in hepatocytes of HFD-metoformin treated group. Representative electron microscopy images (magnification ×15,000; scale bar, 1.0 μm), mark lipid droplets (LP) engulfed by autophagosome. **p < 0.01, ****p < 0.0001, HFD group versus the control group, or metformin + HFD-fed group versus the HFD group.

An autophagy dysregulation may contribute to the pathogenesis of NAFLD (Kwanten et al., 2014). For instance, an impaired autophagic activity in the liver increases lipid accumulation and results in NAFLD (Sinha et al., 2012; Gonzalez-Rodriguez et al., 2014; Martinez-Lopez and Singh, 2015). Therefore, we hypothesized that metformin reduces obesity, hepatic steatosis, and IR, probably *via* the activation of autophagy. To verify the activation of autophagy in the liver, we evaluated the levels of autophagy proteins Atg7, LC3B-II, as well as autophagic substrate p62/SQSTM1 by using Western blotting. We found that the protein expression of LC3B-II was significantly decreased, while the levels of p62/SQSTM1 were increased in the HFD-fed mice (Figure 3A). The metformin treatment significantly reversed the levels of LC3B-II and p62/SQSTM1 (Figure 3A). However, the protein level of Atg7 was not changed in each group (Figure 3A). We further analyzed the liver tissues by using electron microscopy and observed more autophagosomes in the metformin-treated obese mice (Figure 3B). These data demonstrate that metformin promotes autophagy in the liver of HFD-induced NAFLD mice. We also observed that the lipid droplets engulfed by autophagosome in the metformin-treated obese mice (Figure 3C). This evidence of enahnced lipophagy by metformin.

### Metformin Induces Autophagy Through the Activation of TFEB in the Liver of NAFLD Mice

**FIGURE 4:**
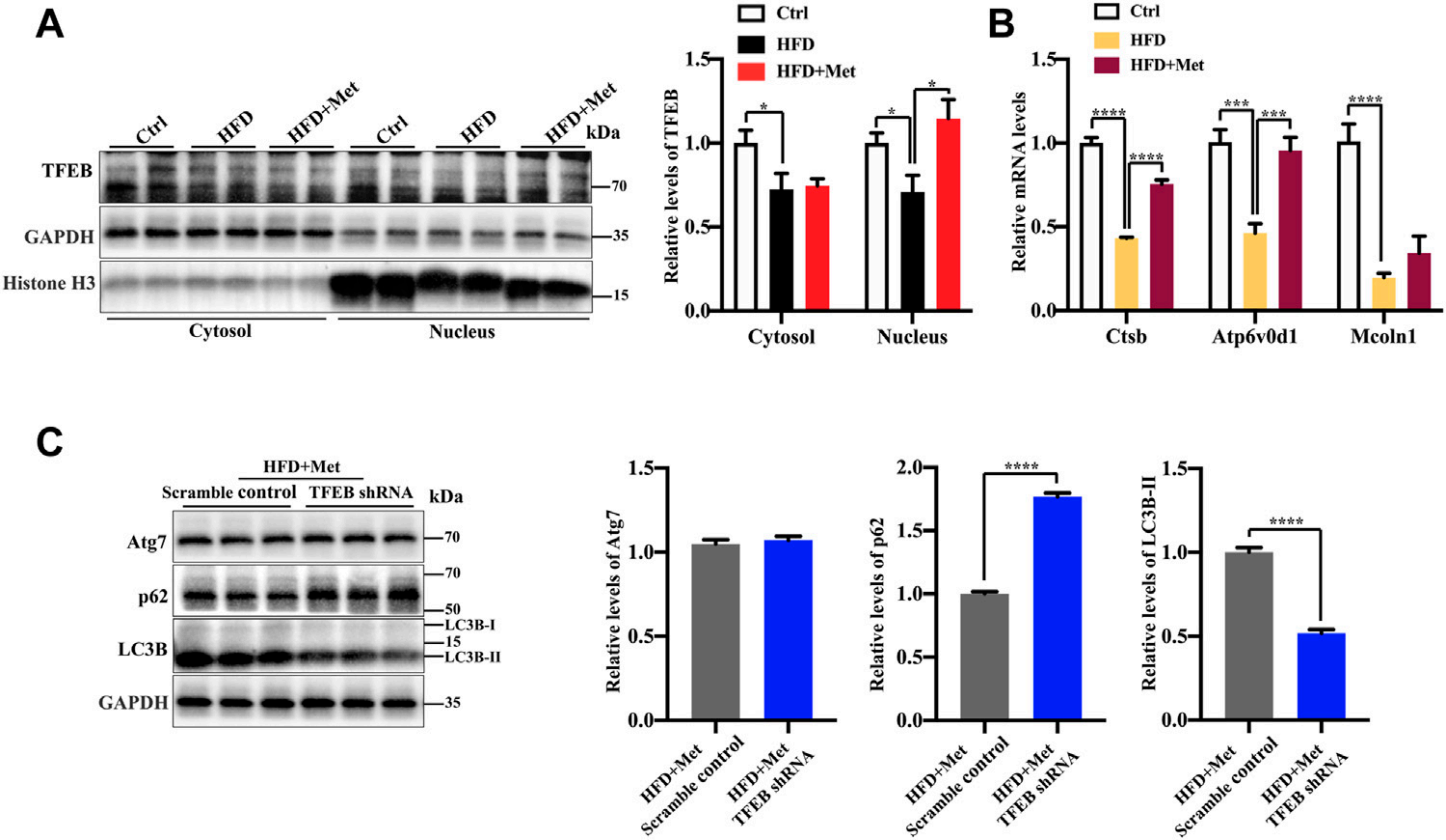
Metformin induces autophagy *via* TFEB in the liver of NAFLD mice. **(A)** Protein levels of TFEB were analyzed using Western blotting. Representative Western blots are shown. **(B)** Quantitative real-time PCR analysis of the TFEB target gene expression. **(C)** Western blotting analysis of Atg7, LC3B-II, and p62 expression in HFD + Met + Scramble control group and in the HFD + Met + TFEB shRNA group. Values are presented as mean ± SEM, *n* = 6 per group. *p < 0.05, ***p < 0.001, ****p < 0.0001, HFD group versus the control group, or metformin + HFD-fed group versus the HFD group, or HFD + Met + TFEB shRNA group versus the HFD + Met + Scramble control group.

TFEB is a major regulator of autophagy and lysosomal biogenesis (Sardiello et al., 2009; Settembre et al., 2011). The activity of TFEB mainly depends on its phosphorylation status and cytoplasm-nucleus shuttling (Puertollano et al., 2018), and the nuclear translocation is a hallmark of TFEB activation. We first examined whether metformin activated TFEB. Using Western blotting, we found that metformin treatment resulted in an increased nuclear accumulation of TFEB (Figure 4A) and reduced phosphorylation of TEFB (Supplementary Figure 1), Furthermore, metformin treatment significantly upregulated the expression of the TFEB target genes, such as those encoding Cathepsin B (CTSB), a member of the lysosomal cathepsin family, which can modulate autophagy processes in adipocytes (Araujo et al., 2018), ATPase H+ transporting V0 subunit D1 (ATP6V0D1), a subunit of the lysosomal proton-transporting V-type ATPase (v-ATPase) (Miles et al., 2017; Jung et al., 2018), which responsible for acidifying intracellular compartments and providing energy required for transport processes of lysosomal, mucolipin 1 (Mcoln1), which regulates lysosomal Ca^2+^ release (Medina et al., 2015) (Figure 4B). These data suggest that TFEB is activated by metformin in the liver of HFD-fed mice.

To further test whether TFEB is involved in metformin-induced autophagy, we performed the knockdown of TFEB *via* tail vein injection with an AAV8 virus expressing an shRNA that targets TFEB (shTFEB). Compared with the injections of a scrambled control shRNA, the injections of recombinant AAV8-shRNA-TFEB reduced the expression of TFEB, as well as that of its target genes (Supplementary Figure 2). Western blotting revealed that the knockdown of TFEB downregulated the expression of LC3B-II and upregulated the protein expression of p62/SQSTM1 (Figure 4C). Likewise, a downregulated TFEB level did not affect the protein level of Atg7. Taken together, these results confirm that metformin induces autophagy through the activation of TFEB.

### Metformin Improves Excessive Lipid Accumulation and IR in HFD-fed Mice *via* TFEB

**FIGURE 5:**
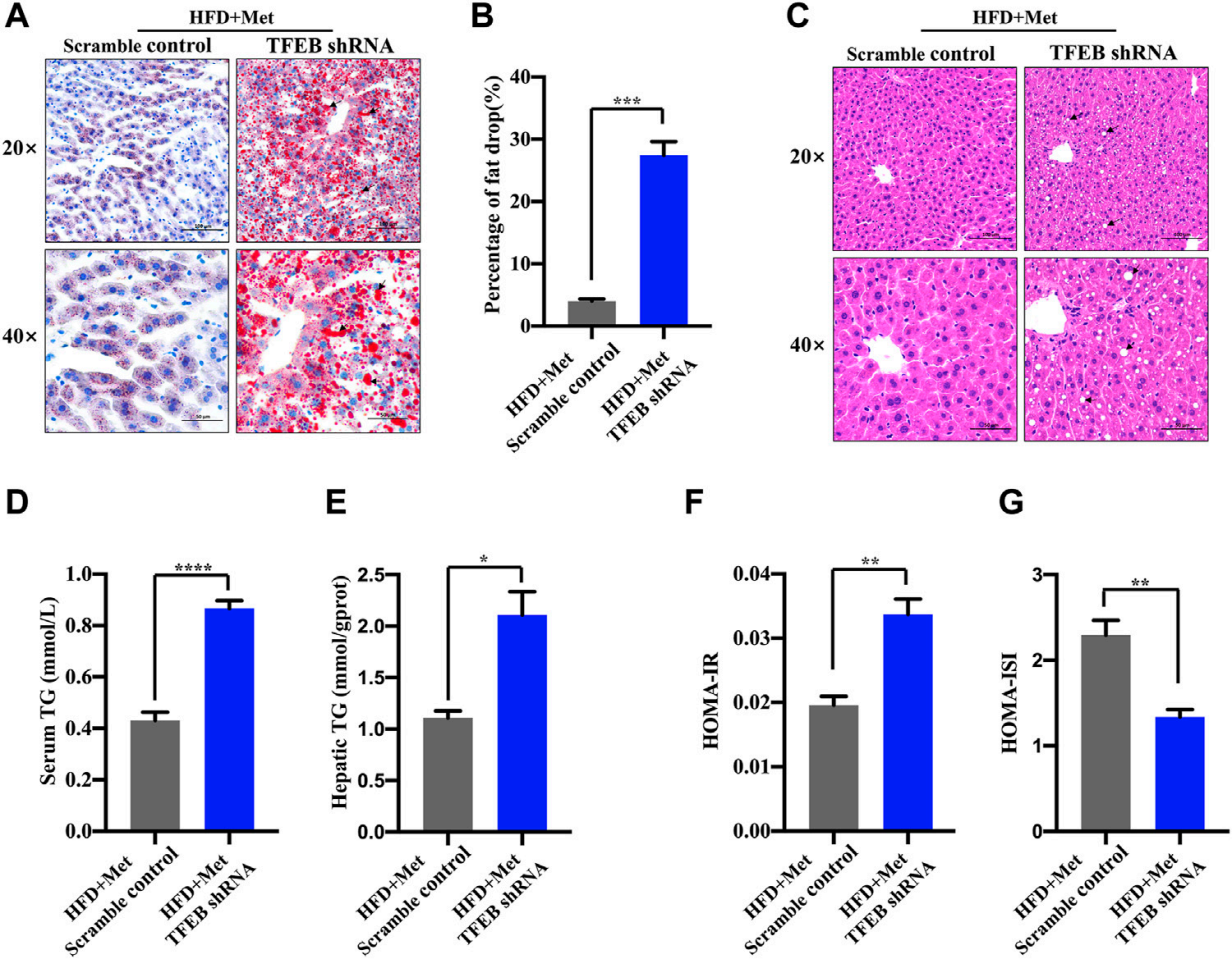
Metformin improves hepatic steatosis and IR *via* TFEB in HFD-fed mice. **(A)** Fat droplets in the liver were detected using Oil red O staining. Representative images of Oil red O staining are shown, where the black arrows denote the fat droplets (magnification ×200 or ×400; scale bar, 50 or 20 μm). **(B)** The percentage of area stained with Oil red O was quantified. **(C)** Representative H&E staining shows the fat vacuoles in the liver. **(D)** The serum and **(E)** hepatic triglyceride (TG) levels were determined in the HFD + Met + Scramble control group and in the HFD + Met + TFEB shRNA group. **(F)** HOMA-IR index and **(G)** HOMA-ISI in each group. Values are presented as mean ± SEM, *n* = 6 per group. *p < 0.05, **p < 0.01, ***p < 0.001, ****p < 0.0001, HFD + Met + TFEB shRNA group versus the HFD + Met + Scramble control group.

To test whether the activation of autophagy mediated by TFEB was involved in the alleviation of an excessive lipid accumulation and IR in HFD-fed mice, we measured the hepatic steatosis level using H&E and Oil red O staining after the knockdown of TFEB. Our data revealed that the accumulation of lipid droplets in the liver was not improved by metformin after the knockdown of TFEB (Figures 5A–C). Moreover, the knockdown of TFEB blocked the effect of metformin on the levels of serum and hepatic TG (Figures 5D,E), increasing the IR index (Figure 5F; Table 2) and decreasing the ISI index (Figure 5G; Table 2) in the mice fed an HFD. Together, these data demonstrate that the protective effects of metformin on excessive lipid accumulation and IR are critically dependent on the TFEB in HFD-fed induced NAFLD mice.

**TABLE 2:** The levels of fasting blood-glucose and insulin to calculate the HOMA-IR and HOMA-ISI after knockdown of TFEB.

Group	Fasting blood-glucose (mmol/L)	Insulin (mIU/L)	HOMA-IR	HOMA-ISI
HM+SC	4.767 ± 0.536	0.095 ± 0.013	0.020 ± 0.001	2.295 ± 0.172
HM+TS	6.375 ± 1.321	0.132 ± 0.024	0.034 ± 0.002^a^	1.336 ± 0.089^a^
*T*	0.989	1.255	4.674	5.389
*P*	0.368	0.265	0.006	0.003

Homeostatic model assessment for insulin resistance (HOMA-IR) index, Homeostatic model assessment for insulin sensitivity index (HOMA-ISI). Data presents mean ± SEM; n = 6.

a p < 0.01, HFD+Met+TFEB shRNA (HM+TS) group versus HFD+Met+Scramble control (HM+SC), respectively, using *t*-test.

## Discussion

**FIGURE 6:**
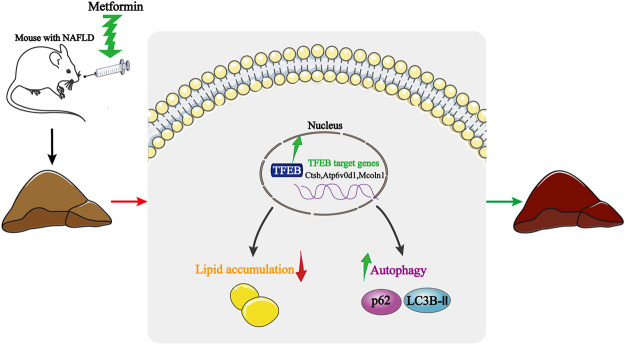
Schematic model of how metformin improves hepatic steatosis, insulin resistance, and autophagy *via* TFEB in NAFLD mice. Metformin activates TFEB in HFD-fed mice, TFEB activation induces autophagy, attenuates insulin resistance, and suppresses an excessive lipid accumulation. Thus, the protective effects of metformin are dependent on TFEB.

NAFLD is characterized by a lipid metabolism collapse, which is often associated with IR, obesity, and metabolic syndrome (Heo et al., 2019). Here, we provide experimental evidence showing that the activation of autophagy is associated with the protective effect of metformin in HFD-fed induced NAFLD mice. Interestingly, the transcriptional activity of TFEB is inhibited in NAFLD, while a metformin treatment dramatically reverses the transcriptional activity of TFEB. Moreover, the activation of autophagy is mediated by TFEB. These findings, therefore, reveal a novel molecular mechanism for improving the NAFLD by using metformin, and highlight the potential beneficial effects of TFEB for the treatment of NAFLD (Figure 6).

Autophagy is widely known as a conserved protein degradation process, which clears dysfunctional organelles and abnormal proteins in a lysosome-dependent manner. In addition to participating in numerous biological events, autophagy also plays an important role in the pathological processes of several diseases, including neurodegenerative diseases, obesity, type 2 diabetes, inflammation, cardiac diseases, aging-related diseases, and cancer (Levine and Kroemer, 2008; Allaire et al., 2019). Accumulating evidence suggests that autophagy contributes to liver homeostasis, and prevents the progression of acute liver injury, nonalcoholic steatohepatitis, and chronic alcohol-related liver disease (Allaire et al., 2019). The hepatic lipid degradation process by autophagy that produces free fatty acids is termed lipophagy (Yang et al., 2010). Lipophagy is involved in regulating energy homeostasis and the intracellular lipid stores. In NAFLD, defective lipophagy occurs at multiple levels through multiple mechanisms and is causally linked to NAFLD-related metabolic disorder (Wu et al., 2018). Numerous studies have demonstrated that an increased autophagy may protect against hepatic steatosis. Hence, autophagy in the liver could serve as a preventive mechanism against NAFLD. For instance, the overexpression of Atg7 in the liver is sufficient to prevent hepatic steatosis in ob/ob mice (Yang et al., 2010). Additionally, pharmacological approaches that restore autophagy also counteract NAFLD-related disease progression (Wu et al., 2018). In contrast, blocking autophagy by silencing the expression of autophagy-related genes or using pharmacological inhibitors exacerbates the pathogenesis of NAFLD (Singh et al., 2009). Mice deficient in Atg7 and Atg14 in hepatocytes or in Atg5 in endothelial cells show a marked increase in endoplasmic reticulum stress, in the accumulation of lipid droplets, and in the production of inflammatory cytokines (Xiong et al., 2012; Liu et al., 2015; Hammoutene et al., 2020). Similar results are observed in animals treated with autophagy inhibitors, including 3-MA and Rubicon (Seglen and Gordon, 1982; Tanaka et al., 2016). In this study, we show that metformin alleviates lipid accumulation and IR in HFD-fed mice, which is accompanied by the activation of autophagy. These data imply that the function of metformin against NAFLD might be associated with the activation of autophagy.

Accumulating evidence confirms that mTOR, SIRT1, phosphatidylinositol 3-kinase (PI3K), extracellular signal-regulated protein kinases (ERK), TFEB, and AMPK are the main regulators of autophagy in lipid metabolic homeostasis (Jung et al., 2009; Cuyas et al., 2018; Allaire et al., 2019; Wang et al., 2019). In the current study, we focused on TFEB, a master regulator of autophagy and lysosomal function. TFEB has important functions in organelle biogenesis and metabolic processes. Recent studies have revealed the underlying mechanisms of TFEB-induced cellular degradative pathways, which in turn promote the clearance of pathogenic factors in a variety of murine disease models (Napolitano and Ballabio, 2016). Furthermore, TFEB plays a positive role in the pathological processes of NAFLD. The activation of TFEB protects the liver from HFD-induced damage by regulating autophagy, lysosomal biogenesis, and fatty acid oxidation (Settembre et al., 2013). Therefore, activators of TFEB might be potential therapeutic candidates for NAFLD. A recent study shows that digoxin, ikarugamycin, and alexidine dihydrochloride may promote TFEB nuclear translocation, thereby inhibiting IR and hepatic steatosis, and enhancing the autophagic flux in mice fed an HFD (Wang et al., 2017). Liraglutide, a glucagon-like peptide-1 receptor (GLP-1R) agonist, attenuates hepatic steatosis *via* the GLP-1R-TFEB-mediated autophagy pathway (Fang et al., 2020). Furthermore, activating TFEB by ezetimibe and tetrahalose can inhibit the NRLP3-dependent IL-1β production and reduce NAFLD lesions (Kim et al., 2017; Sergin et al., 2017). These findings further support the hypothesis that a drug that promotes TFEB is promising for NAFLD treatment.

Metformin was recently shown to induce autophagy *via* TFEB to increase the survival of random pattern skin flaps (Wu et al., 2019). Our studies indicate that a similar TFEB-autophagy-dependent protective effect also exists in the case of HFD-induced NAFLD. In conclusion, the present data suggest that the effects of metformin of attenuating IR and suppressing an excessive hepatic lipid accumulation are dependent on TFEB. Metformin treatment enhances the TFEB-mediated autophagy activity. Hence, our study highlights the potential function of TFEB in the protective effect of metformin in HFD-fed induced NAFLD mice.

## Data Availability

The original contributions presented in the study are included in the article/Supplementary Material, further inquiries can be directed to the corresponding authors.

## Abbreviations

AMPK, 5’-AMP-activated protein kinase; DIO, HFD-induced obesity; HFD, high-fat diet; HOMA-IR, homeostatic model assessment of insulin resistance; HOMA-ISI, homoeostasis model assessment of insulin sensitivity index; IIS, insulin/IGF-1signalling; IR, insulin resistance; Met, metformin; mTOR, mammalian target of rapamycin; NAFLD, nonalcoholic fatty liver disease; SIRT1, NAD-dependent deacetylase sirtuin-1; TFEB, transcription factor EB; TG, triglycerides.
