# Supplementary material for: Metformin Alleviates Hepatic Steatosis and Insulin Resistance in a Mouse Model of High-Fat Diet-Induced Nonalcoholic Fatty Liver Disease by Promoting Transcription Factor EB-Dependent Autophagy
Source: Front Pharmacol. 2021 Jul 23;12:689111. doi: 10.3389/fphar.2021.689111 (PMC8346235; doi:10.3389/fphar.2021.689111)
Supplement: Supplementary file 1 [file Table1.docx]

| **Gene Name** | **Forward** | **Reverse** | **Product Size (bp)** |
| --- | --- | --- | --- |
| Ctsb | ATGACAAGCCTTCCTTCCACC | TAGAAGTTGCGTCCAGCCTGC | 91 |
| Atp6v0d1 | GAGCAGGACCTTGATGAGATGA | GTGCCGAAAGAGTTGATGGTGA | 182 |
| McoLn1 | GGTTATCCTCACCTGCTCCCT | CGAATGACACCGACCCAGA | 297 |

**Supplementary Table 1** The primers used for qRT-PCR assay.
